# Individuals’ Self-Reactions Toward COVID-19 Pandemic in Relation to the Awareness of the Disease, and Psychological Hardiness in Saudi Arabia

**DOI:** 10.3389/fpsyg.2020.588293

**Published:** 2020-12-14

**Authors:** Aljawharh Ibrahim Alsukah, Nourah Abdulrhman Algadheeb, Monira Abdulrahman Almeqren, Fatimah Sayer Alharbi, Rasis Abdullah Alanazi, Amal Abdulrahman Alshehri, Futiem Nasha Alsubie, Reem Khalid Ahajri

**Affiliations:** School of Psychology, Princess Nourah Bint Abdulrahman University, Riyadh, Saudi Arabia

**Keywords:** COVID-19, public health, psychological hardiness, behavior, psychological impact

## Abstract

The coronavirus (COVID-19) outbreak around the world has caused public health concerns and changes in peoples’ behaviors and psychological distress. The pandemic impacts on human behavior, emotions, and cognition, leading to diverse reactions in relation to awareness of the disease. However, there is little understanding around the psychological impacts of the pandemic and strategies to overcome this impact. This study aimed to examine individuals’ reactions toward the COVID-19 pandemic in relation to their psychological hardiness, their degree of awareness toward the pandemic, and precautionary measures taken. Individuals living in Saudi Arabia were invited to complete an online questionnaire which included demographic items, psychological responses to the pandemic, awareness of COVID-19, and measures of psychological hardiness. A total of 1272 individuals were recruited into the study, with the majority being female (85%). Results indicated that the average psychological responses to the COVID-19 pandemic in the study sample were 75.85%. This indicates that the sample generally has a high level of positive psychological responses to the COVID-19 pandemic. The awareness of COVID-19 among Saudi was 91.50%. This indicates a high level of awareness among the study sample.

## Introduction

The coronavirus (COVID-19) pandemic came as a shock to businesses, governments, and individuals. Just as it is threatening people’s health, the pandemic is rapidly becoming a social and economic stressor, as people adopt new living methods to prevent the spread of the disease (Cao and Li, 2020). This pandemic has multiple influences on human behavior, emotions, and cognition, leading to diverse reactions in relation to awareness of the disease (Clay and Parker, 2020). Markedly, the COVID-19 pandemic elicits behavioral and psychological reactions that are likely to result in mental health issues amongst individuals depending on their level of awareness of the disease (Eurosurveillance Editorial Team, 2020; Lee and You, 2020; Lei et al., 2020).

Threats cause unpredictability among people, and they make decisions and choose behaviors that they perceive as useful in regaining a sense of control. Governments across the globe continue to issue facts and precautionary measures to reduce the spread of the pandemic at the individual, community, and international level. Citizens receive this information through media channels and public health channels, which are not evenly accessible. Consequently, people have different knowledge about the disease, its severity, and mortality. Variations in the information provided, lack of enough resources on the condition, and unfamiliarity with its varied outcomes reinforce the perception of its riskiness (Lee and You, 2020). Particularly, the unfamiliarity with the pandemic can heighten people’s perception of its riskiness, which influences the development of behaviors such as hoarding. According to Gyulai (2020), a need to regain control when faced with uncertainties of contagion influences hoarding behaviors. As people seek to gain control, they revert to hoarding items that they perceive to be vital for their survival, including toilet paper, as was witnessed at the peak of the pandemic’s outbreak. Lack of unfamiliarity with the epidemic results in heightened risk perception and risk-averse behavior.

Positive behavioral reactions stem from people’s awareness and optimistic attitudes toward the pandemic. According to Zhong et al. (2020) people’s adoption of safety behaviors, such as the use of preventive masks and hand sanitizers are primarily influenced by their attitudes, practices, and knowledge toward the virus. Socially related behaviors associated with disease awareness include avoiding crowded areas. However, these precautionary measures are reinforced at the state level through strict prevention and control measures such as banning public gatherings (Zhong et al., 2020). A study conducted among the Chinese population indicated that increased knowledge and awareness of the pandemic was associated with a high likelihood of positive attitudes, but potentially dangerous practices toward the epidemic (Zhong et al., 2020). These include closing schools and areas of work, which has contributed to a significant increase in unemployment. Other studies report similar behavioral reactions to the pandemic. For instance, those with little awareness of the illness pretended to be sick to avoid going to their academic or working institutions (Balkhi et al., 2020). Those who are aware of the potential of media channels to increase anxiety levels opted to avoid watching, listening, and reading current news (Balkhi et al., 2020). The positive behavior reactions could result from good knowledge and awareness of the high infectivity rate of the virus.

The COVID-19 pandemic has triggered fear and anxiety in people at some point since it began, regardless of their level of awareness (CDC, 2020). In normal circumstances, misinterpretation of perceived bodily changes and sensations results in health anxiety (Rajkumar, 2020). However, outbreaks of infectious diseases such as the COVID-19 trigger excessive health anxiety, especially when the information provided is exaggerated or inaccurate. Individual anxiety reactions manifest as maladaptive behaviors, such as avoidance of healthcare services, hoarding particular items, and repeated medical consultations, even with the slightest symptoms such as heat-induced headaches. The anxiety further induces mistrust of public authorities regarding the occurrence and preventive recommendations offered (Rajkumar, 2020). Uncertainty, misinformation, and unpredictability of the disease heighten anxiety levels among people, especially those at a high risk of contracting the infection. Zandifar and Badrfam (2020) state that the fear of death among patients is a common reaction related to disease pandemics such as COVID-19. Other likely reactions include the development of anger issues and feelings of loneliness, especially among those quarantined.

People’s reactions to the breakout of COVID-19 have had a huge influence on their mental health, leading to the adoption of self-destructive behaviors among some. As governments continue to impose strict restrictions on movement, people’s livelihoods and routines are disrupted, leading to increased levels of depression, loneliness, and the development of harmful alcohol and drug use, and self-harm.

“Psychological Hardiness” has a great importance in life, as it protects humans from the effects of various life pressures. It is defined as a personality trait with three interrelated dimensions. These dimensions include commitment, which is the tendency to regard life as interesting and meaningful; control, which is a belief that one can influence outcomes by taking action; and finally challenge, an explorative approach to living (Bartone, 2012). It makes the individual more resilient, optimistic and easy to deal with his stressful problems. Psychological hardiness works as a protection against physical illnesses and psychological disturbances. Kobasa (1979) highlights the fact that psychological hardiness and its components work as a psychological variable, alleviating the effect of stressful events on the individual’s physical health. The most hardened people are exposed to stresses and do not get sick. Kobasa agreed with Folkman and Lazarus in that psychological characteristics such as psychological hardiness, for example, affect the individual’s cognitive security to the stressful event itself and the threat comes to his security, mental health and self-esteem. It also affects the individual’s assessment of confrontation methods, which include problems, escaping, avoiding, taking responsibility, seeking support.

On the protective role that personality hardiness plays in protecting an individual from the risk of disease, several studies have been conducted that have found that the trait of hardiness prevents the individual’s level of tendency to deal effectively and logically with stressful situations (Holahan and Moos, 1987; Williams et al., 1992). The significance of the hardiness lies in protecting the individual from disease as it contains the trait of internal control (Kravitz et al., 1993) and the lack of a neurotic factor.

As the COVID-19 pandemic sweeps across the world, it is causing widespread concern, fear and stress, all of which are natural and normal reactions to the changing and uncertain situation that everyone finds themselves in. As Dr. Kluge, WHO regional director for Europe states: “The issue facing each and every one of us is how we manage and react to the stressful situation unfolding so rapidly in our lives and communities. Here we can draw on the remarkable powers of strength and cooperation that we also fortunately possess as humans. And that is what we must try to focus on to respond most effectively to this crisis as individuals, family and community members, friends and colleagues,” (Wang et al., 2020).

Psychological hardiness is the most important characteristic of the personality that grows and develops. It also plays an important role in realizing, facing and resisting stressful events. It has an impact in evaluating the individual’s psychological and social sources to confront pressure. It is a characteristic that may vary from one to another and, sometime at times, the stressful events play an important role in its development and advancement. Psychological hardiness contributes to the emotional, psychological and social maturity to prevent the impact of stressful events on mental and physical health of individuals. Thus, the following research questions are posed in the proposed study:

(1) What is the level of individuals’ self-reaction toward COVID-19 pandemic, degree of the awareness of the disease and the psychological hardiness in Saudi Arabia
(2) Is there a relationship between individuals’ self-reactions toward COVID-19 pandemic in relation to psychological hardiness, in Saudi Arabia?
(3) Is there a difference in individuals’ self-reaction toward COVID-19 epidemic, hypochondria, due to the degree of awareness toward the disease in the Saudi society?
(4) Is there any significant differences in individual’s self-reaction toward COVID-19 epidemic, and the degree of awareness toward the disease in relation to age, gender, marital status, educational level, job type, level of effect of the dieses in Saudi Arabia?

## Materials and Methods

### Study Sample and Methods

An online survey Google form in Arabic was sent to the participants by different social media which they could fill out at their convenience. The first page contained a brief that informed participants about the study protocol, anonymity of data and the right to withdraw at any time during the study. Upon consent, the participants were guided through the questionnaire online. After completion, the participants were presented with a debrief. The questionnaire took up to 20 min to complete, which included the time reading through the briefing and debriefing.

The estimated population of Saudi Arabia (in 2019) is 34.4 million individuals, with 42.6% being female and nearly 47% are within the working age range of 25 and 54 years (Global media insight, 2020). Given that 26.12 million of the population is from the age of 14 and above and calculating a confidence level of 95% with a margin of error of 5% a sample size of 385 was needed. The final sample of the study consisted of (1,272) individuals, age ranged between 13 and 77 years with an average age of (36.85, SD = 12.61). The participants were from 13 regions in Saudi Arabia, distributed as following (1047 in Riyadh, 1 in Al Bahah, in from Al-Jawf, 17 in the northern border; 21 in Qassim, 8 in Medina, 26 in Tabuk, 1 in Jazan, 11 in Ha’il, 17 in Asir, 48 in Mecca, 6 in Najran, 67 in Al Sharqiya). The majority of the sample were female (1083 females by 85%; males = 189, 14.9%). Most of the sample were married (*n* = 771, 60.6%), university-educated (*n* = 809, 63.6%), and more than one third of the sample worked in the educational sector (*n* = 528, 41.5%).

### Measures

#### Psychological Responses to COVID-19

The questionnaire measured individual’s reaction to COVID-19 pandemic, including the cognitive, emotional, social, or behavioral reactions (Supplementary Appendix 1). The questionnaire was developed by the authors and items were created based on open-ended questions sent to a random sample (N 180) in Saudi Arabia. Questions addressed the effects of the pandemic on individuals, their knowledge, emotional, and social behaviors. Participants responses were analyzed, and a total of 68 items were constructed. The questionnaire was sent to six experts in the field of psychology for their feedback. Items were edited according to their suggestions and edits. The final version of questionnaire consisted of (48) items; 23 positive items and 25 negative items. The negative items (reversed) are: (1-2-3-4-5-13-14-15-16-17-18-19-20-21-24-33-41-42-44-45-44).

They are distributed in four constructs, as follows:

1. Cognitive Responses: The set of ideas and beliefs of an individual toward the COVID-19 pandemic; 12 items (1–12).
2. Emotional Responses: The combination of feelings and emotions that the individual feels toward the COVID-19 pandemic, and the number of items of this dimension; 12 items (13–24).
3. Social Responses: Social interactions with others are intended to be the period of the COVID-19 pandemic; 12 items (25–36).
4. Behavioral Responses: The various activities and actions of individuals are intended to be carried out by the COVID-19 period; 12 items (37–48).

The scales were based on a four item Likert scale (completely applicable, completely not applicable) and the scale is corrected by grades (1–4), and the grades are reversed for negative items. Validity was conducted on a random sample of (168) using a factor analysis for the questionnaire items. Results of exploratory analysis revealed that the items statistically significantly are under four factors. These four factors together accounted for 43.88% of the total variance, which is a large amount to reduce the variance explained by these factors.

#### Awareness of COVID-19 Scale

The scale was developed by the researchers based on the information available by the WHO with regard to COVID-19. The scale aimed to measure the extent to which individuals know COVID-19; the nature of the disease, ways of transmission, symptoms, and preventive action, it is consisted of 18 items based on five-point Likert scale (1 = low level of knowledge, 5 = high level of knowledge.).

The scale was sent to six experts in the field of psychology to review the items according to the guidelines presented by the WHO, and changes were made accordingly. The scale was sent to a random sample of (*n* = 168) to test the validity and reliability. The correlation coefficient and cronbach’s alpha was calculated for the statements.

#### Psychological Hardiness Scale

This scale aims to measure individuals’ psychological hardiness, the extent to which the individual perceives in his/her ability to use the available psychological and environmental resources to raise one’s awareness, understanding and effectively face life pressures. The scale was developed by Mukhaimer (2002), consisted of 47 items (32 positive items; 15 negative items).

They are distributed under three constructs, as follows:

1. Commitment: A type of psychological contract that the individual commits to himself, his or her goals, values, and others around him, consisting of 16 items.
2. Control: Indicates how much an individual believes he can have control over the events he receives, and how much personal responsibility is to be assumed for what happens to them, and consists of 15 items.
3. Challenge: The belief that the change in aspects of life is more dramatic and necessary for growth than a challenge to it; this helps it to start and explore the environment and to learn the psychological and social resources that help the individual effectively cope with pressures, consisting of 16 items.

The scale is answered by response alternatives (always, sometimes never), the scale is corrected by scores (1–3) and the scores are reversed for the negative items, and the high scores on the scale reflects high psychological hardiness.

Mukhaimer (2002) tested the scale validity and reliability by calculating the reliability related to the ego strength scale and the reported Cronbach’s Alpha = 75, and compared to Beck depression scale with Cronbach’s Alpha = 0.61 This indicates that both reported Cronbach’s’ Alpha are statistically significant. The reliability of the psychological hardiness scale has been verified in the same ways as the reliability of the reaction feedback scale toward the COVID-19 pandemic.

### Statistical Methods

Several statistical methods were applied to address the research questions. Psychological responses to the pandemic, individuals’ level of awareness and their psychological trauma were calculated using percentages. The correlation coefficient of pearson correlation coefficient, to measure the relationship between psychological and psychological trauma as they are connected variables. The Mann–Whitney test for the two separate samples was conducted to examine the differences in psychological and psychological responses according to the different awareness of the disease. *T*-Tests were conducted for separate sample to examine the differences in psychological responses to the pandemic, based on dimorphological variables that compare two groups (gender, disease not infected). Demographical variables that compare more than two groups (social status, age, qualification, type of occupation) use one-way variation analysis.

## Results

Means and percentages are presented in Table 1. The average psychological responses to the COVID-19 pandemic in a sample of Saudi society were (*M* = 145.64) and the average was 75.85% of the maximum score for the scale (range 144 to 192). This indicates that the sample of research from Saudi society generally has a high level of positive psychological responses to the COVID-19 pandemic. The average degree of awareness of the disease in a sample of Saudi society was (82.35) and the average ratio was 91.50% of the maximum score of the scale, and the average was in the high range (66 to 90). This indicates that the sample of research from Saudi society generally has a high level of awareness of the disease.

**TABLE 1:** Average grade of the sample search on the measure of psychological responses to the COVID-19 pandemic and the average percentage (*n* = 1272).

**Dimensions**	**Average percentage**	**Average**	**Performance levels on metrics**					
			**High**		**Medium**		**Low**	
			**Number**	**Ratio**	**Number**	**Ratio**	**Number**	**Ratio**
Psychological responses to the COVID-19 pandemic	75.85%	145.64	722	56.76%	550	43.24%	–	–
Cognitive responses	82.83%	39.76	1239	97.40%	33	2.60%	–	–
Proactive responses	78.92%	37.88	1070	84.10%	202	15.90%	–	–
Social responses	76.38%	36.66	1089	85.60%	183	14.40%	–	–
Behavioral responses	65.29%	31.34	533	41.90%	736	57.90%	3	0.20%
Awareness of COVID-19 disease	91.50%	82.35	1235	97.09%	37	2.91%	–	–
Psychological hardiness	78.07%	110.08	676	53.14%	592	46.54%	4	0.31%
Commitment	82.69%	39.69	1208	95%	64	5%	–	–
Control	74.33%	33.45	1019	80.10%	249	19.60%	4	0.31%
Challenge	76.96%	36.94	1092	85.80%	180	14.20%	–	–

The average psychological hardiness of a sample of Saudi society was (110.08) and the average ratio was 78.07% of the maximum grade of the scale, and the average is in the high range (from 109.67 to 141). This indicates that the sample of research from Saudi society generally has a high level of psychological hardiness. The highest average psychological hardiness dimension was (commitment), with an average of 39.69 out of 48, followed by second average after (challenge) with an average of (36.94 out of 48), third and final (control) with an average of (33.45 out of 45). These three averages are located at the high level of each of the three dimensions.

### Relationship Between Psychological Responses to COVID-19 and Psychological Hardiness

There is a statistically significant positive correlation (at 0.01) between all dimensions (cognitive, emotional, social, and behavioral) and the overall degree of psychological responses toward the pandemic (Table 2). The higher the psychological responses toward the pandemic in terms of dimensions and overall grade, the higher the scores of the psychological response the higher the psychological hardness, and vice versa.

**TABLE 2:** The correlation coefficient between the psychological responses of individuals to the COVID-19 pandemic and, and psychological hardiness, in a sample of Saudi society (*n* = 1272).

**Variables**	**Cognitive**	**Eminency**	**Social**	**Behavioral**	**The overall degree of psychological responses to the COVID-19 pandemic**
Commitment	0.21**	0.30**	0.34**	0.34**	0.45**
Control	0.15**	0.27**	0.19**	0.29**	0.35**
Challenge	0.18**	0.28**	0.27**	0.26**	0.37**
The overall degree of psychological trauma	0.22**	0.35**	0.33**	0.36**	0.48**

**Statistically Significant at (0.01) level.

The grades of the sample of the disease awareness were high and only at the high and intermediate levels, depending on the degree of the levels of the variables in the search measures shown in Table 3. A statistically significant difference (at 0.01) between average grades of individuals with a high level of awareness of the disease in the psychological responses of individuals to the COVID-19 pandemic in favor of an average grade of high-awareness. Individuals with a high level of awareness of the disease have positive psychological responses to the COVID-19 pandemic, in statistical terms, then their mid-level counterparts.

**TABLE 3:** Mann–Whitney test examining differences in psychological responses to COVID-19 pandemic, and psychological hardiness, according to the degree of awareness of the disease.

	**Levels**	***N***	**Mean rank**	**Sum of rank**	***U* value**	**Significance level**
Psychological responses of individuals to the COVID-19 pandemic	Medium	37	322.57	11935	5.28	0.01
	High	1235	645.91	797693		
Psychological hardiness	Medium	37	490.96	18165.5	2.45	0.05
	High	1235	640.86	791462.5		

The statistically significant difference (at 0.05) between average grades of individuals with high level of mental strength and high awareness of the disease in favor of average grades of high level of disease awareness. Individuals with a higher level of awareness of the disease have higher psychological hardness than their peers with a higher level of awareness of the moderate disease.

### Relationship Between Awareness of COVID-19 and Psychological Responses to the Pandemic

There was statistically little variability in the average male and female scores in the psychological responses to the COVID-19 pandemic (Table 4). However, there was a statistically significant difference in individuals’ psychological responses to the COVID-19 pandemic when the type of disease infection was considered in a sample from the Saudi society (Table 5). There is a convergence between the average degrees of injured and uninjured in the psychological responses to the COVID-19 pandemic.

**TABLE 4:** *T*-test, one-way contrast analysis and the Mann–Whitney Tests examining differences in the psychological responses to the COVID-19 pandemic across demographic variables.

	***N***	**Mean (SD)**	**Value (*t*)**	**Significance level**
**Gender**				
Female	189	146.2 (12.18)	0.66	0.51
Male	1083	145.54 (12.56)		Change a function
**Social status**				
Single	404	141.9 (13.05)	27.84	0.01
Married	771	147.28 (11.65)		
Absolute or widows	97	148.15 (13.44)		
**Age**				
Less than 30	394	140.95 (12.96)	39.48	0.01
From 30 to less than 40	315	145.76 (11.33)		
From 40 to less than 50	320	147 (11.93)		
50+	243	151.28 (11.13)		
**Educational level**				
Minor and lower	179	146.42 (12.33)	7.61	0.01
University	809	144.66 (12.59)		
Higher than University	284	147.92 (12.08)		
**Type of occupation**				
Employed in the education sector	528	148.34 (12.13)	16.31	0.01
Health Officer	48	144.21 (13)		
Employed in other sectors	137	144.88 (11.9)		
Student	293	141.23 (13.09)		
Does not work	266	145.79 (11.37)		

**TABLE 5:** Differences in the psychological responses to the COVID-19 pandemic type of disease.

**Type of disease**	***N***	**Mean**	**Total grades**	**Value (*z*)**	**Significance level**
Not injured	1242	635.52	789317.5	0.61	0.54
Injured or recovering	30	677.02	20310.5		Change a function

There was a statistically significant differences (at 0.01) in individuals’ psychological responses to the COVID-19 pandemic between social statuses. The positive psychological responses of married, divorced, widowed, and widowed were higher than in those who were single. There was a statistically significant differences (at 0.01) in the psychological responses of individuals to the COVID-19 pandemic across the age groups. Individuals who were in the 50 years and older group were found to be higher in statistical terms in the psychological responses to the COVID-19 pandemic compared to all ages under 50. There was a statistically significant differences (at 0.01) in the psychological responses of individuals to the COVID-19 pandemic in terms of educational level. The higher-level education (higher than university) was found to be statistically higher in the psychological responses to the COVID-19 pandemic compared to the higher (university) level.

Staff in the education sector were also to be found to be higher in statistical terms in the psychological responses to the COVID-19 pandemic compared to employees in other sectors, non-employees and students.

## Discussion

The aim of this study was to examine the psychological impact of COVID-19 in Saudi Arabia. Awareness about COVID 19 among Saudi was extremely high, which may be due to when the study was conducted. Due to the experience of the epidemic of MERS (Middle East respiratory syndrome related coronavirus) in 2015 in Saudi Arabia, the population were more aware of COVID-19 virus diseases. Zhong et al. (2020) indicated that the knowledge score of Chinese about the COVID-19 was similarly high to what our study found (90%). Although Saudi Arabia was largely unaffected by MERS, public knowledge of MERS at that time was high, 79.35% (Althobaity et al., 2017), which is less than the recorded knowledge of COVID-19 (86.5%) (Hoda, 2016). However, Bawazir et al. (2018) indicated that almost all participants heard about the (MERS) COVID-19 disease and causative agent however the overall knowledge was 66.0%. The reason of the difference between the results of the studies may be the time of data collection in relation to the spread of the disease.

A study done by Wolf et al. (2020) in the United States to investigate the knowledge of COVID 19 showed that participants who had heard of COVID-19 considered the disease to be a high threat. However, Clements (2020) reported that COVID-19 knowledge in the United States was approximately 80%, which was lower than the 91.5% reported in our study. Today most people in Saudi Arabia have easy access to the internet and information from different sources which could explain the high level of knowledge among people in Saudi Arabia.

Illness anxiety disorder (previously called hypochondriasis) is described as a person who worries that he\she may become seriously ill, or they may have serious disease. Gong et al. (2020) reported that hypochondriacal suspicion was 44.11%. Our results showed that the COVID-19 pandemic brought panic and hypochondria to the public, with just under half of the sample thinking they had the disease. Eichenberg and Schott (2019) reported that more than 40% of their Austrian participants showed at least some symptoms of hypochondria. Wolf et al. (2020) reported that 24.6% said that they were “very worried” about getting coronavirus. Huang and Zhao (2020) reported that anxiety about COVID-19 in China was 35.1%. AlNajjar et al. (2014) reported that 57.7% of Saudi Arabians recorded a moderate anxiety score about MERS, due to the higher mortality rates of MERS in comparison to COVID-19. In addition, the animal suspected of carrying the MERS virus was a camel, which is a common animal in Saudi Arabia.

In term of hardiness, we did find that hardiness levels in our sample was 78.07%. Brooks (2003) reported that higher levels of hardiness correlate with more positive outcomes in chronic illness patients. As hardiness increases, the positive outcome of the disease increases and in our study we did find that as awareness increased, hardiness increased. Costantini et al. (1997) reported that nurses working in Oncology or AIDS patient who have higher hardiness levels at the beginning of the course of treatment were associated with lower emotional exhaustion and higher personal achievement. One factor to be considered in hardiness was spiritual well-being. Carson and Green (1992) reported that there was a significant relationship between spiritual well-being and hardiness. People in Saudi Arabia have strong relationships to their religion which could be explained for the high levels of hardiness in our study during the COVID-19 pandemic.

We did find that 75.85% of our sample have a psychological impact due to the pandemic of COVID 19. The young have higher psychological impact however male and female have the same level of psychological impact. In contrast Varshney et al. reported that in India one third of respondents had significant psychological impact, younger age, female gender and comorbid physical illness have higher psychological impact. Wang et al. (2020) examined the psychological impact of COVID-19 and reported minimal psychological impact, mild psychological impact and moderate or severe psychological impact to be 24.5, 21.7, and 53.8% respectively.

Coronavirus is a serious disease and have shown that people are anxious about the disease. All governments have taken a major step to slow the spread of the virus. Mortality rates as of September 2020 in Saudi Arabia is less than 1% (Google search, 2020). However, the mortality rate of MERS at Saudi Arabia was reported at 37 and 22.9%. In the South Korea this was 21%. It is very clear that the MERS disease carries a higher mortality rate than COVID-19. It is very clear that psychological impact in the public is great. Further and more detailed studies to evaluate the best solution to combat devastating psychological impact is needed.

## Data Availability Statement

The raw data supporting the conclusions of this article will be made available by the authors, without undue reservation.

## Ethics Statement

The studies involving human participants were reviewed and approved by Princess Nourah Bint Abdulrahman University Ethics Committee. The patients/participants provided their written informed consent to participate in this study.

## Author Contributions

All authors who meet authorship criteria are listed each contribution. Furthermore, each author certifies that this material or similar material has not been and will not be submitted to or published in any other publication. AAA designed the analysis. NA and MA contributed to analysis. FSA, AAA, and RKA wrote the literature review. RKA performed the analysis. FSA collected the data.

## Conflict of Interest

The authors declare that the research was conducted in the absence of any commercial or financial relationships that could be construed as a potential conflict of interest.
