# Corrigendum: Individuals' Self-Reactions Toward COVID-19 Pandemic in Relation to the Awareness of the Disease, and Psychological Hardiness in Saudi Arabia

**DOI:** 10.3389/fpsyg.2021.703589

**Published:** 2021-05-26

**Authors:** Aljawharh Ibrahim Alsukah, Nourah Abdulrhman Algadheeb, Monira Abdulrahman Almeqren, Fatimah Sayer Alharbi, Rasis Abdullah Alanazi, Amal Abdulrahman Alshehri, Futiem Nasha Alsubie, Reem Khalid Ahajri

**Affiliations:** School of Psychology, Princess Nourah Bint Abdulrahman University, Riyadh, Saudi Arabia

**Keywords:** COVID-19, public health, psychological hardiness, behavior, psychological impact

There were errors in the Funding statement. The corrected funding statement is below:

This research was funded by the Deputyship for Research & Innovation, Ministry of Education in Saudi Arabia. The project number is PNU-DRI- Targeted-20-019.

The authors apologize for this error and state that this does not change the scientific conclusions of the article in any way. The original article has been updated.

