# Supplementary material for: Individuals’ Self-Reactions Toward COVID-19 Pandemic in Relation to the Awareness of the Disease, and Psychological Hardiness in Saudi Arabia
Source: Front Psychol. 2020 Dec 14;11:588293. doi: 10.3389/fpsyg.2020.588293 (PMC7767923; doi:10.3389/fpsyg.2020.588293)
Supplement: Supplementary file 1 [file Data_Sheet_1.docx]

Appendix 1 Questionnaire

**Self-Reactions Scale Against COVID-19**

Greetings,

The world has experienced Covid-19 pandemic, may Allah Almighty protect all, and have had a number of psychological and social impacts upon us. Through this study, the Saudi Association for Psychological Counseling at Princess Noura Bint Abdul Rahman University aims to monitor the most important self-reactions of individuals towards this pandemic, whether emotional, cognitive, social, or behavioral reactions.

In supporting of scientific research in our dear country, we hope you will kindly cooperate by answering the scale in its four parts, noting that answering the scale will not take you more than 15 minutes, Allah willing. There are no right or wrong answers. The correct answer is what about you in this period more than others.

There is no need to provide your name, your information will be treated confidentially and will be used for scientific research purposes only.

We appreciate your cooperation and your responseTop of Form

Part I

The following statements represent the extent of your knowledge about Covid-19. Please select the option that represents your knowledge of information from among the five-stage gradual (1-5), where number (1) represents low knowledge, and number (5) high knowledge.

1.Have you followed the statistics of COVID-19 around the world or in your region (injuries, recovery, deaths): *

Option 1 (I never follow)

Option 2

Option 3

Option 4

Option 5 (I closely follow)

2. COVID-19 is airborne as transmitted through droplets that are dispersed from the mouth or nose during coughing and sneezing: *

Option 1 (I don't know)

Option 2

Option 3

Option 4

Option 5 (totally agree)

3. COVID-19 is transmitted through contact with surfaces contaminated with the Virus: *

Option 1 (I don't know)

Option 2

Option 3

Option 4

Option 5 (totally agree)

4. COVID-19 is transmitted by close contact with others: *

Option 1 (I don't know)

Option 2

Option 3

Option 4

Option 5 (totally agree)

5. The most common symptoms of COVID-19 as follow: High temperature, fatigue, and dry cough: *

Option 1 (I don't know)

Option 2

Option 3

Option 4

Option 5 (totally agree)

6. Among the severe symptoms of COVID-19 are difficulty breathing: *

Option 1 (I don't know)

Option 2

Option 3

Option 4

Option 5 (totally agree)

7. The incubation period of COVID-19, i.e. the period from infection with the virus to the onset of symptoms of the disease, ranges between one and 14 days, and it usually lasts five days: *

Option 1 (I don't know)

Option 2

Option 3

Option 4

Option 5 (totally agree)

8. Are you satisfied with the amount of health information available about COVID-19: *

Option 1 (Not at all satisfied)

Option 2

Option 3

Option 4

Option 5 (Fully satisfied)

9. Level of confidence in doctors' ability to diagnose or recognize symptoms associated with COVID-19: *

Option # 1 (Not at all confident)

Option 2

Option 3

Option 4

Option 5 (Very confident)

10. The probability of survival when infected with COVID-19: *

Option 1 (Unlikely at all)

Option 2

Option 3

Option 4

Option 5 (Very likely)

11. The main source of health information on COVID-19: *

Option # 1 (Friends and family)

Option 2

Option 3

Option 4

Option 5 (Official sources of information)

12. I consider the Ministry of Health's instructions to stay at home: *

Option # 1 (Not interested)

Option 2

Option 3

Option 4

Option 5 (Very committed)

13. I make sure to cover the mouth and nose when coughing or sneezing with the bent elbow or a tissue, and then dispose of the used tissue immediately: *

Option # 1 (Not interested)

Option 2

Option 3

Option 4

Option 5 (Very careful)

14. I make sure to wash may hands with soap and water frequently for at least 20 seconds: *

Option # 1 (Not interested)

Option 2

Option 3

Option 4

Option 5 (Very careful)

15. I put on the mask when I have symptoms of COVID-19 (especially cough), or if I am caring for someone who may have this Virus: *

Option 1 (I don't know)

Option 2

Option 3

Option 4

Option 5 (I put on in the recommended conditions)

16. I sanitize purchases, and dispose of shopping bags immediately when I get home: *

Option 1 (Not interested)

Option 2

Option 3

Option 4

Option 5 (Very careful)

17. I make sure to frequently clean the house with disinfectants and sterilizers, especially the surfaces exposed to touch such as door handles and faucets: *

Option 1 (Not interested)

Option 2

Option 3

Option 4

Option 5 (Very careful)

18. I contact the competent authorities when I or a family member feel temperature rising or have any symptoms: *

Option # 1 (I don't tell anyone)

Option 2

Option 3

Option 4

Option 5 (I consider to communicate)

Part II:

You are required to determine how far the following statements apply to you among the options (completely applies to me, applies to me, does not apply to me, does not apply to me at all), noting that there are no right or wrong answers. The correct answer is what about you in this period more than others.

1- I think that COVID-19 is a big lie. *

Completely applies to me

Applies to me

It does not apply to me

It does not apply to me at all

2. COVID-19 appears to be the end of life. *

Completely applies to me

Applies to me

It does not apply to me

It does not apply to me at all

3. COVID-19 is a biological warfare. *

Completely applies to me

Applies to me

It does not apply to me

It does not apply to me at all

4. COVID-19 is an affliction from Allah due to default of worshipers. *

Completely applies to me

Applies to me

It does not apply to me

It does not apply to me at all

5. I will get COVID-19 virus inevitably. *

Completely applies to me

Applies to me

It does not apply to me

It does not apply to me at all

6. COVID-19 is a global pandemic that has no cure/serum yet. *

Completely applies to me

Applies to me

It does not apply to me

It does not apply to me at all

7. COVID-19 is a period and will pass, Allah willing. *

Completely applies to me

Applies to me

It does not apply to me

It does not apply to me at all

8. COVID-19 is like all other viruses, but it is rapid deployable. *

Completely applies to me

Applies to me

It does not apply to me

It does not apply to me at all

9. I am confident that scientists will find a cure for COVID-19. *

Completely applies to me

Applies to me

It does not apply to me

It does not apply to me at all

10. Even if I get Coronavirus, I will be cured of it, Allah willing. *

Completely applies to me

Applies to me

It does not apply to me

It does not apply to me at all

11. COVID-19 pandemic has united efforts worldwide. *

Completely applies to me

Applies to me

It does not apply to me

It does not apply to me at all

12. COVID-19 has given the earth planet the opportunity to get rid of pollution. *

Completely applies to me

Applies to me

It does not apply to me

It does not apply to me at all

13. I feel sad all the time. *

Completely applies to me

Applies to me

It does not apply to me

It does not apply to me at all

14. I am pessimistic about the mysterious future of COVID-19. *

Completely applies to me

Applies to me

It does not apply to me

It does not apply to me at all

15. I'm concerned about what will happen in the future because of this COVID-19 pandemic. *

Completely applies to me

Applies to me

It does not apply to me

It does not apply to me at all

16. Whenever I remember COVID-19, I feel fear and my heartbeat increases. *

Completely applies to me

Applies to me

It does not apply to me

It does not apply to me at all

17. When I hear the news about COVID-19, I feel pain in my stomach. *

Completely applies to me

Applies to me

It does not apply to me

It does not apply to me at all

18. I find it difficult to breathe when I think I might get COVID-19. *

Completely applies to me

Applies to me

It does not apply to me

It does not apply to me at all

19. I feel I will fail my studies or my career while these events continue. *

Completely applies to me

Applies to me

It does not apply to me

It does not apply to me at all

20. I feel guilty for not following all the precautionary instructions. *

Completely applies to me

Applies to me

It does not apply to me

It does not apply to me at all

21. I often feel nothing (my feelings are dysfunctional). *

Completely applies to me

Applies to me

It does not apply to me

It does not apply to me at all

22. I feel really uncomfortable when I remember the current situation. *

Completely applies to me

Applies to me

It does not apply to me

It does not apply to me at all

23. I feel sympathy for some patients and health practitioners. *

Completely applies to me

Applies to me

It does not apply to me

It does not apply to me at all

24. I am terrified about the rapid spread of the COVID-19, and the increasing number of deaths. *

Completely applies to me

Applies to me

It does not apply to me

It does not apply to me at all

25-i missed my family and friend during quarantine

Completely applies to me

Applies to me

It does not apply to me

It does not apply to me at all

26-life is repetive and boring without interacting with people

Completely applies to me

Applies to me

It does not apply to me

It does not apply to me at all

27- I miss the way my life was begore cerfew

Completely applies to me

Applies to me

It does not apply to me

It does not apply to me at all

28- this crisis relief me from social meeting

Completely applies to me

Applies to me

It does not apply to me

It does not apply to me at all

29- quarantine made family bonds closer

Completely applies to me

Applies to me

It does not apply to me

It does not apply to me at all

30- quarantine helped me rethinking my relationships

Completely applies to me

Applies to me

It does not apply to me

It does not apply to me at all

31- my social life wasn’t affected because I communicate by technology

Completely applies to me

Applies to me

It does not apply to me

It does not apply to me at all

32-during this pandemic I lost my care for others

Completely applies to me

Applies to me

It does not apply to me

It does not apply to me at all

33-i currently cut off relationships with others

Completely applies to me

Applies to me

It does not apply to me

It does not apply to me at all

34- I take initiative in any volunteer work that serves society in this pandemic

Completely applies to me

Applies to me

It does not apply to me

It does not apply to me at all

35- I mentally relief my family and people around me

Completely applies to me

Applies to me

It does not apply to me

It does not apply to me at all

36-I cooperate with my family on chores

Completely applies to me

Applies to me

It does not apply to me

It does not apply to me at all

37- I spend most of my time sleeping

Completely applies to me

Applies to me

It does not apply to me

It does not apply to me at all

38-i passionately watch the news to know updates

Completely applies to me

Applies to me

It does not apply to me

It does not apply to me at all

39-i exercise way more than before

Completely applies to me

Applies to me

It does not apply to me

It does not apply to me at all

40- I spend most of my time finishing my school or work

Completely applies to me

Applies to me

It does not apply to me

It does not apply to me at all

41-i spend a lot of time on social media

Completely applies to me

Applies to me

It does not apply to me

It does not apply to me at all

42-i spend along hours watching shows and movies

Completely applies to me

Applies to me

It does not apply to me

It does not apply to me at all

43-i buy more food during quarantine then ever

Completely applies to me

Applies to me

It does not apply to me

It does not apply to me at all44- I eat more than usual

45- I feel lazy and tired most of the time

Completely applies to me

Applies to me

It does not apply to me

It does not apply to me at all

46- I started to care more about self-care this pandemic

Completely applies to me

Applies to me

It does not apply to me

It does not apply to me at all47- I started to maintain my religion practices more

48- I can't keep track of the days because they are so repetitive

Completely applies to me

Applies to me

It does not apply to me

It does not apply to me at all

Part III:

Read the following statements, and answer with yes (if they apply to you) or no (if they do not apply to you).

1. Sometimes, I get bouts of nausea and vomiting. *

Yes

No

2. I get a heartburn that bothers me several days a week. *

Yes

No

3. I have disturbed sleeping and sleepless. *

Yes

No

4. In some parts of my body, I often feel what feels like burning, chills, numbness or anesthesia. *

Yes

No

5. I get stomach pains every few days. *

Yes

No

6. Most of the time, I feel like my head is about to explode. *

Yes

No

7. I often feel intense pressure around my head. *

Yes

No

8. My stomach often tires me. *

Yes

No

9. Sometimes, I feel that the top of my head is soft*

Yes

No

10. Most of the time, I feel generally weak. *

Yes

No

11. I lose sensation in one or more areas of my skin. *

Yes

No

12. My appetite is good. *

Yes

No

13. Most of days, I wake up active and rested. *

Yes

No

14. My hands and feet are usually warm. *

Yes

No

15. My ability to work has not change than ever before. *

Yes

No

16. A little bit if feel constipated. *

Yes

No

17. My physical health is like the health of most of my friends in terms of quality. *

Yes

No

18. I hardly have any pain in the heart or chest. *

Yes

No

19. I did not have any difficulty controlling the bowel movement. *

Yes

No

20. It almost never occurred to me to feel pain in the back of my neck. *

Yes

No

21. I do not complain of muscle cramps, and if I complain, I rarely do. *

Yes

No

22. I never vomited blood or coughed up blood. *

Yes

No

23. Over the past few years, my health has been generally good. *

Yes

No

24. My weight does not increase or decrease. *

Yes

No

25- I do not get sick quickly

Yes

No

26- I rarely get dizzy

Yes

No

27-i can read for long time without my eye hurting

Yes

No

28- I rarely get headache

Yes

No

29-i have not found difficulty in walks

Yes

No

30-i never felt papilation or shortness in breath

Yes

No

31-i never suffer from pain or rarely

Yes

No

32-my eyesight hasn’t weakened over the years

Yes

No

33-i don’t feel ringing in my ear ever

Yes

No

Part IV and The Finial

You are required to determine how far the following statements apply to you among the options (always / sometimes / never) noting that there are no right or wrong answers.

1. Whatever the obstacles, I can achieve my goals *

Always

Sometimes

Never

2. I make my own decisions and no external source tells me what to do. *

Always

Sometimes

Never

3. I believe that the joy and excitement of life lies in the ability of the individual to face his challenges. *

Always

Sometimes

Never

4. The value of life lies in the individual's loyalty to some principles and values. *

Always

Sometimes

Never

5. When making my future plans, I am often sure I can achieve them. *

Always

Sometimes

Never

6. I break into troubles to fix, not waiting for it to happen. *

Always

Sometimes

Never

7. Most of my life is wasted in meaningless activities.  *

Always

Sometimes

Never

8. My success in my affairs (work - study ... etc) depends on my efforts and not on luck or chance. *

Always

Sometimes

Never

9. I have curiosity and a desire to know what I do not know. *

Always

Sometimes

Never

10. I believe that my life is a purpose and what I live for. *

Always

Sometimes

Never

11. Life is an opportunity, not work and struggle. *

Always

Sometimes

Never

12. I think that an exciting life is which has problems that I can face. *

Always

Sometimes

Never

13. I have certain values ​​and principles that I adhere to and maintain. *

Always

Sometimes

Never

14. I think the failure is due to reasons that lie within every individual. *

Always

Sometimes

Never

15. I have the ability to persevere until I finish any problem I am facing. *

Always

Sometimes

Never

16. I have no goals that I should stick to or defend them. *

Always

Sometimes

Never

17. I think almost everything that happens to me is the result of my choices. *

Always

Sometimes

Never

18. Problems mobilize my strengths and abilities to challenge. *

Always

Sometimes

Never

19. I do not hesitate to participate in any activity that serves the community in which I live. *

Always

Sometimes

Never

20. There is actually no such thing as luck. *

Always

Sometimes

Never

21. I feel fear and threatened due to circumstances and events that may happen to my life. *

Always

Sometimes

Never

22. I take the initiative to assist others when they faced any problem*

Always

Sometimes

Never

23. I think that chance and luck play an important role in my life. *

Always

Sometimes

Never

24-when I solve a problem I am excited to solve more

Always

Sometimes

Never

25-i believe that staying away from people is better

Always

Sometimes

Never

26-i control how my life goes

Always

Sometimes

Never

27- I believe facing problem are test or how much I can handle and perseverance

Always

Sometimes

Never

28-thinking about myself does not gave me a chance to think about other thing

Always

Sometimes

Never

29- I believe bad luck come from bad planning

Always

Sometimes

Never

30- I love adventure and exploring

Always

Sometimes

Never

31-i participate on anything that I think helps my family and society

Always

Sometimes

Never

32-i think I'm weak and can't change that happen to me

Always

Sometimes

Never

33-i face my problems because I believes I can solve them

Always

Sometimes

Never

34- I care about what happens around me from issues and events

Always

Sometimes

Never

35-i believe the lives of individuals is affected by an external force with no controls

Always

Sometimes

Never

36- life that is peaceful and quiet is the best life for me

Always

Sometimes

Never

37- life is not worth living

Always

Sometimes

Never

38- I believe that luck is better than hard work

Always

Sometimes

Never

39-i believe life that does not have change is a boring and repetitive is life

Always

Sometimes

Never

40- I feel responsible for others and offer to help

Always

Sometimes

Never

41-i believe I have a strong effect on what happens around me

Always

Sometimes

Never

42-im scare of change because it might threat on my life

Always

Sometimes

Never

43-i care about my country and involve in in it whenever I can

Always

Sometimes

Never

44-i plan everything and don’t leave it to luck, fate, and coincidences

Always

Sometimes

Never

45-the change is life and the important is to how to force it

Always

Sometimes

Never

46-i change my values and what I believe if I must to change it

Always

Sometimes

Never

47- I feel scare to face problem before it is happening

Always

Sometimes

Never
